# Ayers on Diseases of Rectum

**Published:** 1884-09

**Authors:** Mortimer Ayers

**Affiliations:** Rushville, Ill


### AYERS ON DISEASES OF THE RECTUM.

In the July number of THE HOMŒOPATHIC PHYSICIAN, in a review of my little book on *Some of the Diseases of the Rectum, their Homœopathic and Surgical Treatment*, you say: "Under the above title Dr. Ayers treats of rectal abscesses, ulcers, polypi, hæmorrhoids, fistula in ano, etc., and, curiously enough, he includes constipation as among the diseases of the rectum. Why not include diarrhœa as well? The indications for such remedies as are mentioned for the various diseases are not well stated, and to our mind too *many* topical applications are advised."

As you will notice by the preface, this work was urged upon the author, and as an honest physician he gave for each disease the best treatment that he knew. (Could he do more?) He recognized that the treatment was susceptible of improvement, hence his request for the practical experience of others. The indications for the remedies were presented in that form which the experience of the author had taught him to recognize, at least he has found them reliable. If they are not the best, the reviewer could not serve the cause better than to "state the best" indications for the various remedies in the diseases mentioned, *e. g.*, pruritus, fissure or irritable ulcer, fistula or polypus, to say nothing of sanguineous, capillary, or cutaneous hæmorrhoids.

Constipation was cited so often as one cause of rectal diseases that at the urgent request of several eminent physicians a chapter on Constipation was added. Diarrhœa is not so often a cause of rectal disease, at least in the observation of the author, and hence was not added, besides, there are some good books on diarrhœa.

Theoretically, all diseases of the rectum ought perhaps to be cured by the homœopathic remedy without *any topical applications*, but practically we have not yet been given a remedy or remedies that will cure stricture of the rectum, nor even fistula, hence topical applications and surgical measures are necessarily resorted to, and fortunately do relieve the distressed patient.

Again we say, by all means give us not only the best indications, but any indications for any homœopathic remedy that will cure these annoying persistent rectal diseases, and no one will receive them more gladly than the author.

It might interest other readers of THE HOMŒOPATHIC PHYSICIAN to know which of the many (few) topical applications given in the work (under protest) the reviewer would retain. But until we all come to the fullness of the stature of a *perfectly* informed homœopathic physician let us do what we can to aid the march of Homœopathy, never forgetting that this should guide us always to relieve our patient as much as possible where we cannot completely cure.

MORTIMER AYERS,
Rushville, Ill.

NOTE BY THE EDITOR.—In all reviews of works sent us we have endeavored to give a candid, impartial opinion as to their value to the homœopathic practitioner.

Now, as to Dr. Ayers' book, we have nothing whatever to do with the reasons which led to its publication, nor is it for us to judge of the author's ability or knowledge. We can only state our opinion as to the work *done*. In this book Dr. Ayers' prescriptions and indications are chiefly *local* or topical. He prescribes for fistula or for stricture, and *not for the patient suffering with one or the other of these troubles!!*

Dr. Ayers shows *his* idea of a homœopathic prescription when he writes: "* * * We have not yet been given a remedy or remedies that will cure stricture of the rectum, nor even fistula; hence (!) topical applications and surgical measures are necessarily (!) resorted to, and, fortunately, do relieve (?) the distressed patient."

To this we reply that fistulæ *have been cured* by Arsenic, Aloes, Berberis, Hydrastis, Thuja, Lachesis. These cures we have seen on record, and there are, doubtless, numbers of others. Dr. Dudgeon states that he has cured "several bad cases." Every *homœopath* knows that ulcers, pruritus ani, fissures, etc., can and are cured by internal administration of the dynamized remedy. But one must prescribe for the *patient*, not for the disease.

